# ‘We DECide optimized’ - training nursing home staff in shared decision-making skills for advance care planning conversations in dementia care: protocol of a pretest-posttest cluster randomized trial

**DOI:** 10.1186/s12877-019-1044-z

**Published:** 2019-02-04

**Authors:** Bart Goossens, Aline Sevenants, Anja Declercq, Chantal Van Audenhove

**Affiliations:** LUCAS, KU Leuven, Minderbroedersstraat 8, 3000 Leuven, Belgium

**Keywords:** Advance care planning, Communication, Dementia, Intervention, Nursing homes, Shared decision-making

## Abstract

**Background:**

Due to the gradual loss of function, it is crucial for persons with dementia to discuss advance care planning in due course. However, nursing home staff, residents and their families feel uncomfortable to start this type of conversation, resulting in unknown (care) preferences. ‘We DECide optimized’ will provide tools to nursing home staff for discussing advance care planning. The primary objective is to enhance the level of shared decision-making in advance care planning conversations. We hypothesize that the training will enhance the perception of the importance, competence and frequency in which participants engage in advance care planning conversations. The secondary objective is to assess barriers and facilitators in the implementation of advance care planning policies at the ward level.

**Methods:**

‘We DECide optimized’ will consist of two four-hour workshops and a homework assignment between sessions. Training components will include information on advance care planning and shared decision-making, role-play exercises and group discussions on implementation barriers at the ward level. Participating wards will receive supporting materials to stimulate residents and their families to initiate conversations. The study uses a cluster randomized controlled design, with 65 Flemish nursing home wards taking part (311 staff members). Data will be collected through a pretest-posttest model, with measurements up to 9 months after training. The RE-AIM framework will be used to evaluate the effectiveness of the implementation. Quantitative and qualitative data at the clinical, organizational and resident level will be collected.

**Discussion:**

This study describes a hands-on, in-depth and multi-level training approach to improve shared decision-making in advance care planning conversations. By providing tools to ward staff, engaging the management and informing residents and their families, ‘We DECide optimized’ aims to decrease evidence-based barriers and to provide all stakeholders with incentives to engage in conversations about (care) preferences in an informative and participatory manner.

**Electronic supplementary material:**

The online version of this article (10.1186/s12877-019-1044-z) contains supplementary material, which is available to authorized users.

## Background

Approximately 50 million people worldwide live with dementia [1]. In Flanders (Belgium), 114,000 persons are affected by the syndrome, and this figure is expected to double by 2060 [2]. 65.9% of people with dementia above the age of 65 die in a nursing home [3, 4]. Due to the gradual loss of (cognitive) function and the fact that approximately 71% of persons with advanced stages of dementia die within 6 months of admission, it is imperative that nursing homes record a person’s (care) preferences in a timely and well-documented manner [5, 6]. Advance care planning enables this policy.

Advance care planning (ACP) allows individuals to define goals and preferences for future medical treatment and care, to discuss these goals and preferences with family and health-care providers, and to record and review these preferences as and where appropriate [7]. Involving persons and their families in the decision-making of care preferences is considered a pivotal element in person-centered care. [8]. However, a 2014 nationwide retrospective study reported that only 11.8% of persons with dementia expressed their wishes regarding end-of-life care in nursing homes [9]. Both nursing home staff and residents and their families express apprehensions in initiating ACP. Health professionals usually refrain from taking responsibility because there is no task agreement on ACP at either the ward or the organizational level [10]. They also find it difficult to converse with people living with dementia about their (end-of-life) care preferences [11]. Meanwhile, residents and families lack information on ACP and its benefits [12].

Communication regarding care preferences is also rarely patient driven in nursing homes, which acts as an additional barrier to engage in ACP [9, 13]. This ‘paternalistic’ approach runs contrary to the principles of shared decision-making (SDM), which require the active involvement of persons and their families in care and treatment decisions [14]. The three-talk model for SDM as proposed by Glyn Elwyn and colleagues translates the theoretical concept into a guided approach for clinical practice [15]. The three steps are: (1) making sure that people know that multiple options are available (Choice Talk); (2) providing detailed information about these choices (Option Talk); and (3) considering preferences and working towards a decision (Decision Talk). A recent revision substitutes Choice Talk with Team Talk to depict better the process of collaboration and deliberation between conversation parties [16].

Based on the barriers listed above, Kononovas & McGee propose 4 steps to improve the quality and uptake of ACP [17]. These are: (1) taking steps to inform and empower patients; (2) putting better systems in place; (3) taking responsibility for initiating discussions; and (4) ensuring health professionals have the right skills. Training care professionals in the use of SDM, while simultaneously engaging management, residents and their families, can thus serve as a means to facilitate ACP [11, 18]. It is however necessary that all those involved acknowledge the complex and time pressured environment in which nursing homes operate [19]. Taking these considerations into account, we developed ‘We DECide’ (We Discuss End-of-life Choices), a communication training program for nursing home staff to facilitate SDM in ACP conversations with residents and their families. The three-talk model for SDM guided the development and assessment of the intervention.

A feasibility study was conducted with ‘We DECide’ between 2010 and 2014 in 19 Flemish nursing wards [20]. Focusing on training in communication skills and empowering nursing home staff from different backgrounds to initiate ACP discussions, the intervention showed promising results in improving the competence of participants when using SDM, but did not improve the actual use of SDM in observed conversations [21, 22]. Based on qualitative feedback from participants and on recent literature, additional focus on the organizational level of ACP and the involvement of all SDM stakeholders, including residents and their families, was added to the training [23]. There were no changes to existing training components, though some training materials which were found to be too time-consuming, were reworked into more accessible editions. The revised intervention was named ‘We DECide optimized’.

### Aims

The primary goal of ‘We DECide optimized’ is to enhance the level of SDM in ACP conversations. In order to achieve this goal, we set 4 objectives. (1) At the clinical level, participants need to receive training in communication skills to enhance the frequency and competence with which they use SDM. (2) Participants from all backgrounds need to be invited to the training program, including management and non-healthcare professionals, in order to engage all professionals of the ward in initiating ACP. (3) At the organizational level, wards need to be given the opportunity and guidance to reflect on their ACP policy, including the use of SDM. (4) At the resident level, persons with dementia and their families need to be enticed to engage in ACP discussions and be given the tools to do so.

The secondary goal of ‘We DECide optimized’ is to assess barriers and facilitators in the long-term implementation of ACP policies based on the use of SDM in nursing homes. By engaging professionals both at the clinical and organizational level, as well as the residents themselves, we will receive quantitative and qualitative feedback from multiple stakeholders to improve person-centered care as well as future interventions. Since advance care planning is both an individual and a team-based effort, we opted for a cluster randomized controlled design to assess objectives at both the participant and the ward level.

Our hypotheses are as follows. ‘We DECide optimized’ will increase the level of SDM in ACP discussions. The training will enhance the perception of the importance, competence and frequency in which participants engage in ACP conversations. After a period of 3–9 months of implementing what was learned during the training, these changes will be firmly integrated at the ward level and into the general policy. Both staff and management play an important role in stimulating these changes, with the latter actively supporting the implementation and the use of SDM in ACP discussions after undergoing the training. Residents and their families will feel more involved in expressing their wishes and preferences through our information campaign (see below).

## Methods

### Intervention design

‘We DECide optimized’ is a training program for nursing home staff. Nursing homes participate per ward with both professionals and members of management. The training will consist of 2 workshops of 4 h each, with a homework assignment between sessions. Two trainers, one with a background in psychology and the other in nursing, and each with over 25 years of career experience in holding SDM & ACP conversations in nursing homes, will conduct the training sessions. One of the authors (BG) will be present as well to oversee the research component of the intervention.

**Fig. 1:**
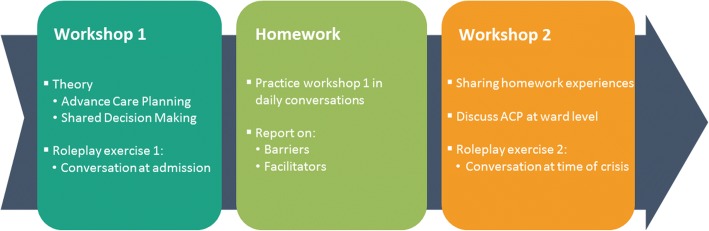
Intervention modules

The training will consist of 3 modules which pertain to both the participant and the ward level. These are: (1) a theoretical introduction to ACP & SDM, (2) role play exercises and (3) reflecting on ACP policy. All wards will also receive supporting materials to stimulate residents and their families in participating in ACP conversations at the end of the first workshop. These modules are described below (Fig. 1. Intervention modules).

### Workshop 1

Participants will first receive a theoretical introduction on SDM and ACP which outlines the concepts and addresses several misconceptions, such as ACP being only a part of palliative care. The importance of a multi-level approach at the clinical, organizational and resident levels will be stressed. Due to the differing backgrounds of our participants, it is important to establish a common ground from which to work on. This module will take about 1 hour.

**Fig. 2:**
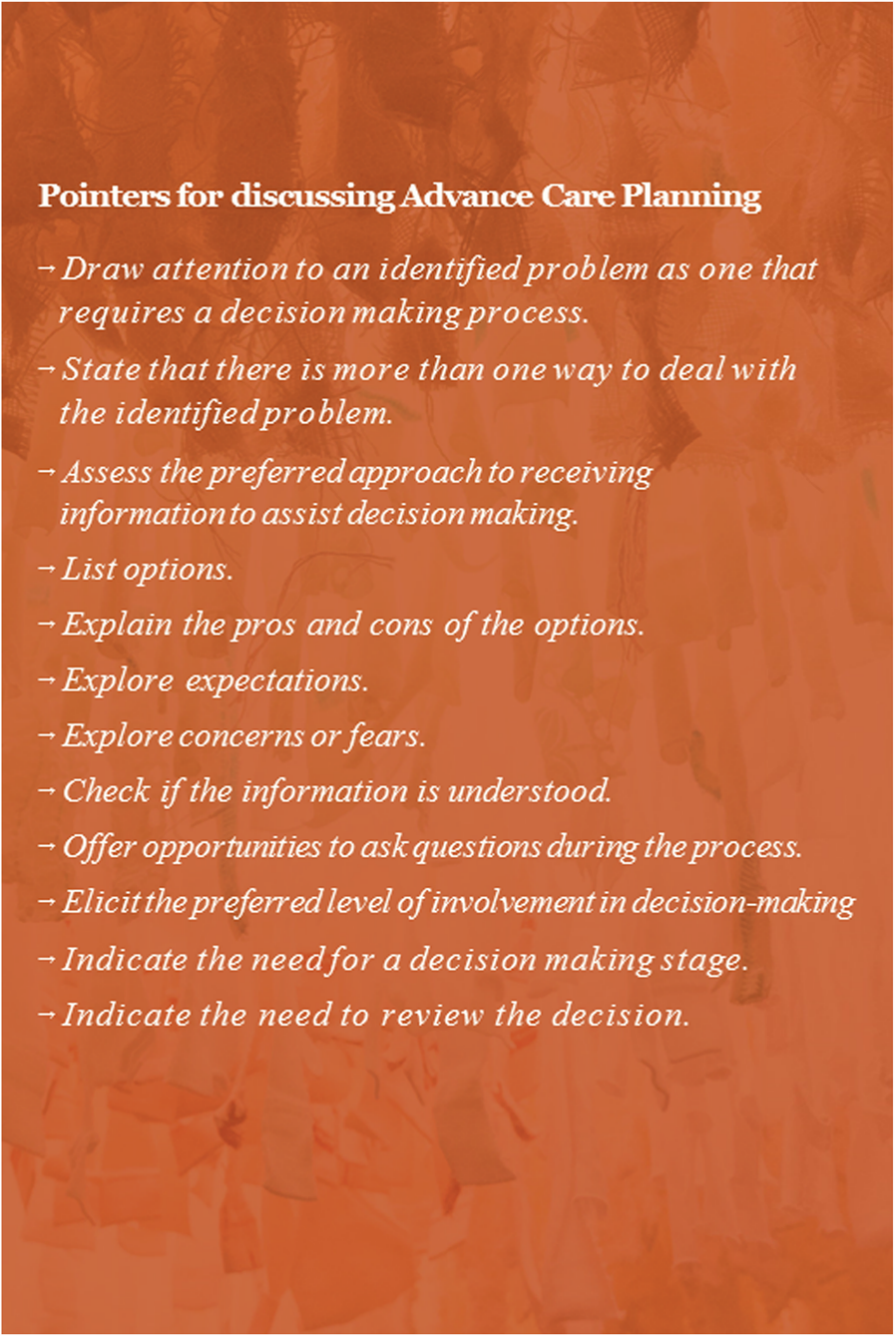
Pointers for discussing Advance Care Planning

We will then introduce the main focus of the training, namely Elwyn’s three-step talk model and discuss the importance of a structured approach to ACP discussions based on SDM [15]. To facilitate the uptake of this information, all participants will be issued a brief card with 12 pointers which summarize Choice Talk, Option Talk and Decision Talk (Fig. 2. Pointers for discussing ACP). After the introduction and discussion of this training method, a role-play exercise will be introduced to begin hands-on training. It is important to note that each role-play exercise in ‘We DECide optimized’ focuses on a critical moment to discuss ACP: at the time of admission, during daily informal conversations and during crisis situations. Since the focus on certain conversation elements changes depending on the time of conversation (working towards a decision will be more important during crisis situations than during the time of admission, for instance), participants will practice the three-step talk model in different situations. The first role-play exercise will focus on a conversation at the time of admission. Participants will take part in the exercise by either re-enacting a case, or by fulfilling predefined observation tasks. Each case will include the role of a person with dementia to ensure that the conversations are tailored to their needs. All cases, roles and tasks for the exercises were adopted from ‘We DECide’ [20]. The trainer will guide the exercise by means of the brief card and will address communication pitfalls as well. At the end there will be a discussion on lessons learned. This portion of the workshop will take about two and a half hours with a brief pause in between. The final half hour of the workshop will introduce information about the homework assignment as well as the information campaign. Wards will also receive a poster of the three-talk step model to introduce and discuss with other colleagues. All materials are designed to be easily disseminated.

### Homework assignment

In the 4 weeks between the two workshops, participants will practice the different steps of the three-step talk model by engaging in at least one informal conversation with a resident living with dementia and/or family. It is important to note that residents or families often implicitly or explicitly express preferences or fears concerning the daily routine in the nursing home ward, for example when another resident becomes ill. Continuously observing and taking these opportunities to talk are important for keeping abreast of the concerns and wishes of the ward population. These moments can also serve as a signal for a formal ACP conversation. Participants are instructed to write down their experiences and list a number of barriers and facilitators which might have hindered or helped the conversation. These homework cases will then be sent in 1 week before workshop 2, during which they will be summarized by the trainer and then discussed during workshop 2. Since this discussion will contain a summary of all barriers and facilitators which were encountered by the group as a whole, the training will not be affected if a participant was unable to do the homework assignment. Reasons for not being able to complete the assignment will be noted, though the participant will not be barred from workshop 2.

### Workshop 2

The homework assignments will be discussed during the first hour of the second workshop. Participants will reflect on their conversations and offer each other suggestions on how to improve their communication skills. This information will be noted by BG as part of his task to oversee the research component of the intervention. Another role play exercise will be introduced during the second hour. It will focus on ACP conversations in crisis situations. If participants have not re-enacted a case during workshop 1, they will be invited to take up a role. At the end, there will be a discussion about lessons learned. Both segments are to be assisted by the trainer. After a short break, participants will be invited to sit down with their other staff members to discuss their ACP policy. They will be asked to list a number of opportunities and ways to improve their policy in a period of 3 to 9 months. Recommendations to achieve person-centered care will be given on a single page document to assist them in this task. This document is based on the ACP Guidelines which were previously used in ‘We DECide’, but were found to be too cumbersome and time-consuming [20]. After 1 hour, both wards will come together again to discuss their opportunities for improvement. The trainer and other ward members will give recommendations for improving the achievability of these ideas. The wards will be contacted in 3 and in 9 months to check progress on their objectives and to provide further incentives for improving their policy. This discussion will last 30 min. During the last half hour, participants will fill in the IFC-SDM questionnaire, which assesses the competence, importance and frequency at which staff members engage in SDM (see also: IFC-SDM). They will also fill in an evaluation form concerning their experiences during both workshops and the homework assignment. Details on post-training measurements and follow-up interviews will be discussed.

### Supporting materials

- *Presentation.* Both workshops are guided by a PowerPoint presentation.
- *Brief card.* Lists 12 OPTION-based pointers for the use of SDM in ACP conversations.
- *A4 document.* Lists 10 recommendations to achieve person-centered care.

### Information campaign

In order to stimulate residents with dementia and their families to participate in ACP conversations, we developed an information campaign in collaboration with the nursing home Huis Perrekes. The title of the campaign is: ‘Shared Decision-Making. Your choice, our care.’ Participating wards will receive multiple copies of these supporting materials at the end of the first workshop. They will be able to provide feedback during the second workshop and during the telephone interviews in 3 and 9 months. Feedback from residents and their families will be gathered at the same time by means of ad hoc forms. The materials are:

### Poster

Two A2-size posters depicting the title of the campaign and the image *Heaven* will be given to participating wards to hang up at key locations, such as the elevator or cafeteria. *Heaven* features a collective knot work of bleached, re-used textiles in different colors. It is made by a collective of residents, children and volunteers to underscore that ACP is a team effort. The poster also invites residents and their families to participate in SDM by talking to their caregivers about it. The image and text are restricted to a minimum in order to not confound persons with dementia or their families with too much information. It is intended to raise questions and initiate conversations.

### Pocket card

30 A6-size pocket cards will be distributed to each ward. These pocket cards prepare residents and their families for an ACP conversation by inviting them to ask 3 questions: (1) What are my options? (2) What are the (dis)advantages of every option? (3) What do I need to make a decision? These 3 questions were adopted from the corresponding article by Shepherd et al. [24].

### Information page

30 A4-size information pages will be handed out to each ward. The pages contain information about SDM, including the three-step talk model, and invite residents and families to reflect upon their care preferences and to request information actively. They are designed to work in tandem with the pocket cards, but can also be used exclusively. Both the information pages and the pocket cards can be put to use in different ways, e.g. to provide information upon admission, in preparation of a formal ACP conversation or as part of an ACP awareness campaign. In this way, we make sure the material can be operationalized in nursing homes with different cultures and approaches to information distribution.

### Study design

**Fig. 3:**
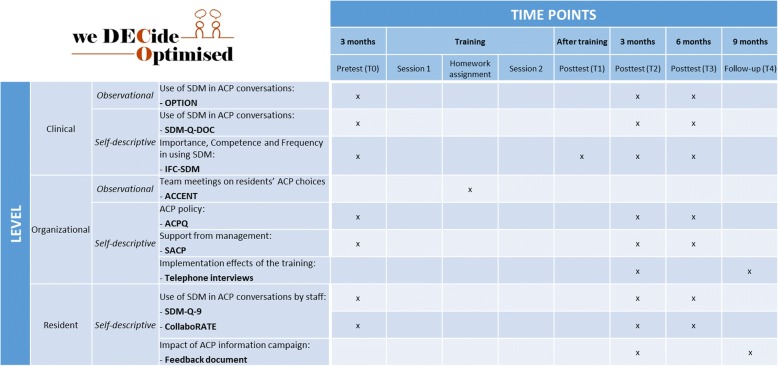
Study overview: time points, levels of measurement and assessment instruments

The study uses a cluster randomized controlled design, with clusters at the ward level. ‘We DECide optimized’ will be implemented in the intervention group, while the control group receives no training. After all measurements have been simultaneously conducted in both groups, excluding the interviews regarding long-term implementation results, the control group will receive training as well. Data will be collected through a pretest-posttest model, with measurements at 5 time points. These are (T0) 3 months prior to training, (T1) directly after the last training session, (T2) 3 months after training, (T3) 6 months after training and (T4) 9 months after training (Fig. 3. Study overview).

This study will report pretest-posttest results by means of the RE-AIM Framework [25]. This model can be used to plan and evaluate public health interventions through the assessment of 5 dimensions: Reach, Effectiveness, Adoption, Implementation and Maintenance (Table 1. RE-AIM dimensions). These dimensions can occur at multiple levels, such as the clinical or organizational level, and thus corresponds to the current study. It is also specifically useful for interventions such as ‘We DECide optimized’, which focus on behavioral change, the assessment of implementation barriers and facilitators and external validity [26].

**Table 1:** RE-AIM dimensions [26]

Dimension	
Reach	Number, percentage and representativeness of patients who participated in the intervention.
Effectiveness	Intervention effects on targeted outcomes.
Adoption	Number, percentage and representativeness of participating settings and providers.
Implementation	The extent to which the intervention was consistently implemented by staff members.
Maintenance	The extent to which the intervention becomes part of routine organizational practices, and maintains effectiveness.

### Required sample size

The required sample size is calculated through the clusterPower software package for R, which takes the cluster-randomized design of the study into account [27]. Using a power of 0.80, assuming unequal cluster sizes (x̄=5, σ_X_ = 1) and an intraclass correlation of 0, a sample of 32 nursing home wards per condition is needed at the 5% significance level.

### Settings/participants

Participation calls were sent out by e-mail to the 4 major umbrella organizations for nursing homes. To maximize sample size, each of the 755 Flemish nursing homes was also individually contacted by means of a flyer. Enrollment was voluntarily, albeit 5 inclusion criteria needed to be met. These were: (1) The ward unit must be focused on persons with dementia, or at least have a mixed population. (2) A minimum of 4 to a maximum of 6 ward staff members can participate. (3) At least one of these members must stem from either middle or executive management. This person must be directly involved with the participating ward (e.g. work there or coordinate tasks) and delegate all information and assessment requirements to the other members. These other participants can be either care or non-care professionals as long as they interact with the residents and their families on a regular basis. (4) Enlisting wards enroll in both the training as well as the research module. (5) The nursing home has not participated in ‘We DECide’, and will not participate in other ACP research for the duration of the training.

One hundred ninety nursing homes responded to the flyer, whereupon they received more information. Forty-eight of these 190 nursing homes subscribed for a total of 65 wards (311 participants). The primary reasons for not participating in the study were resistance from management or staff members, reluctance to send in audio files (see also: OPTION) and the long-term duration of the intervention. We also received a lot of interest from psychiatric wards, which we unfortunately had to decline because the intervention was not tailored to the specific population.

BG distributed the 65 nursing home wards in the intervention and control group through simple randomization in Excel before any measurements were conducted. The randomization was conducted at the nursing home level (48) at a 1:1 ratio to avoid contamination between clusters in a single nursing home. This resulted in 34 wards in the intervention group and 31 wards in the control group. Clusters, participants and trainers were blinded of group assignment (single-blind trial). Participants completed written informed consent forms upon enrollment before the randomization.

### Outcome measurements

#### Primary outcomes

Our primary outcome is the use of SDM in formal ACP conversations. This outcome is measured at both the participant and the resident level through 3 instruments, which are selected based on their usage in comparable (inter)national research and with regard to their psychometric properties. Both observational and self-descriptive instruments are used, since the triangulation of data is considered especially important to report on effective change management in an adequate manner. For measurements on the resident level, brief and easily accessible questionnaires are chosen so as not to overburden the person with dementia or his or her family. See also: Fig. 3. Study overview*.*

- OPTION-12 is a valid and reliable observational instrument which measures the involvement of patients by clinicians during consultations (α = 0.79) [28, 29]. Observers score 12 items on SDM on a five-point Likert scale ranging from ‘the behavior is not observed’ to ‘the behavior is exhibited to a very high standard’. In this study, we use the Dutch translation to assess the extent to which nursing home staff involve residents and their families during formal ACP discussions. Participating wards send in 2 audio files of formal ACP conversations at the pretest stage as well as 3 and 6 months after training. These audio files are recorded by the same participants at the different time points. Recordings are rated by two blind researchers. Both read through the OPTION training manual beforehand. During the course of the feasibility study, OPTION-5 was developed [30, 31]. Though the latter differentiates better between various levels of patient involvement, both show good interagreement and correlate well with each other [32]. For this reason and so as not to differentiate from the feasibility study, we continue to use OPTION-12 during the course of this study.
- The ‘Shared Decision Making Questionnaire’ (SDM-Q) is an internationally validated questionnaire which assesses the implementation of SDM in clinical conversations. It can be used to investigate the effectiveness of interventions which aim to increase said level of SDM [33]. It consists of 9 items which are rated on a six-point Likert scale ranging from ‘completely disagree’ to ‘completely agree’. It is filled in separately by both the healthcare professional (SDM-Q-DOC) and the patient (SDM-Q-9) to assess their view on the conversation. The instrument was translated and validated in Dutch (SDM-Q-DOC α = 0.87, SDM-Q-9 α = 0.88) [34]. For this study, the SDM-Q was used in conjunction with the OPTION audio files to compare data from both instruments. Residents and their families received a pre-stamped envelope which they can use to send the SDM-Q-9 to us without any involvement from the nursing home ward.
- The Dutch variant of collaboRATE is used to assess further the view of residents on their involvement in ACP conversations. [35] This questionnaire gauges the level of SDM in 3 items on a 10-point Likert-scale, ranging from ‘no effort was made’ to ‘every effort was made’. Both are discriminative as concurrent validity to other instruments, including the SDM-Q, is established [36]. For this study, collaboRATE is filled in at the same time as the SDM-Q and sent in the same pre-stamped envelope. Due to the limited amount of items, it should also be more accessible in situations where residents and families consider the SDM-Q to be too burdensome.

#### Secondary outcomes

Our secondary outcomes provide more insight into the mechanisms which might facilitate the use of SDM in ACP conversations. These include the view of participants on their competence, a measurement of the actual ACP policy at the ward level, and support from management. All secondary outcomes are measured by means of self-report questionnaires.

- The IFC-SDM measures nine behavioral aspects needed to engage in SDM and is based on the three-step talk model [16]. Each aspect is measured on three scales: how important participants find this behavior; how frequently they put this behavior into practice; and how competent they feel in doing so. To distinguish between the use of SDM in different work settings, the questionnaire assesses these behaviors in three circumstances: for conversations at the time of admission, during daily practice and in crisis situations. Items are scored on a 5-point Likert scale, ranging from ‘not important at all’ to ‘extremely important’. Participants can also indicate that they do not know. The IFC-SDM is based on earlier work by our research group and was pilot tested before the feasibility study [20]. No changes were made afterwards. The questionnaire is completed in the pretest stage, immediately after the last training session and after 3 and 6 months.
- The ‘Advance Care Planning Questionnaire’ (ACPQ) measures the organization of ACP at the ward level. 45 questions assess how and with whom ACP is discussed at the time of admission, in a crisis situation and at the start of palliative care. Answers are then compared with practice guidelines for communication on end-of-life issues for persons with dementia in nursing homes and scored either 0 or 1 for a total of 45 points. The higher the score, the more the guideline criteria are met. The ACPQ was piloted during the feasibility study in the form of a group audit [20]. This method was found to be too time-consuming considering the current sample size. It also did not allow for differentiating scores between care professionals and management staff. For this reason, the audit was turned into a questionnaire. One item was rewritten to reflect the underlying question better. The ACP is filled in by all participants at the pretest stage and after 3 and 6 months.
- The self-constructed ‘Supporting Advance Care Planning’ (SACP) assesses the support from management in discussing ACP and optimizing its organization (see Additional files 1 & 2). Participants answer eight items on two scales: how important they find the item and how much it is implemented at the ward level. These eight questions are based on the barriers which were most frequently mentioned at the end of the feasibility study. The items are scored on a 5-point Likert scale, ranging from ‘not important at all’ to ‘extremely important’. The questionnaire is filled in the pretest stage and after 3 and 6 months.

### Additional and control measures

- Telephone interviews at 3 and 9 months after the last training session gauge participants’ progress in implementing the training components at the clinical, organizational and resident level (see Additional files 3 & 4). Barriers and facilitators are discussed, including recommendations for future research. A standardized form is created to ensure the interviews are conducted in a similar manner.
- Wards record a team meeting on ACP between training sessions. This requirement is part of a cross-sectional study which analyzes the correlation between the use of SDM in ACP conversations and the manner in which ACP is discussed during team meetings. The self-constructed instrument ‘Assessing Care Choices of the Elderly in Nursing home Teams’ (ACCENT) scores the audio files on 5 questions by means of a 5-point Likert scale, ranging from ‘*totally not agree’* to ‘*totally agree*’ (see Additional files 5 & 6). The questions are based on OPTION and pertain to the manner in which elicited care preferences are treated and followed-up, as well as the involvement of all team members in discussing the subject. The latter reflects our view that ACP should be a team-based effort.
- The following demographic variables of participants are measured: age, gender, educational status, profession and employee tenure. We also record if discussing ACP is part of their routine and if they received any previous training on SDM.

### Statistical analysis

Due to the cluster-randomized design of the study, longitudinal multilevel analysis (linear mixed modeling with random intercepts at both the clinical and the ward level) is used as the method of analysis. Since nursing homes can participate in the training with multiple wards, an additional organizational level is added as well. SPSS 25 is used for the analysis.

First, the effectiveness of ‘We DECide optimized’ on the primary outcome – the use of SDM in ACP conversations – is assessed. Second, the effectiveness of the training on the secondary outcomes – (1) self-perceived importance, competence and the use of SDM in ACP conversations, (2) ACP policy and (3) support from management – is examined. Separate analyses assess whether our demographic variables influence the primary outcome. Only significant predictors are added to the final model.

Differences in baseline characteristics between the intervention and control group are analyzed by descriptive analysis.

## Discussion

This article deals with the design of a cluster-randomized controlled trial which measures the effectiveness of ‘We DECide optimized’, a communication training program for nursing home staff, on the use of SDM in formal ACP conversations. Due to the complex nature of dementia, it is crucial for residents and their families to be able to express their preferences and wishes in an informed and timely manner. Furthermore, both clinicians and residents hesitate to initiate conversations due to a lack of information and experience. ‘We DECide optimized’ addresses both concerns by training nursing home staff in the use of SDM: participants receive information on SDM and ACP and engage in various role-play exercises with help from an experienced trainer. One strength of this training is the inclusion of professionals from all backgrounds to engage the ward as a whole in discussing ACP. Furthermore, each ward delegates one member of management to stimulate the implementation of the training at the ward level. An additional strength of the training is our multi-level approach: by engaging the clinical, organizational and resident level, all stakeholders in ACP are invited to immerse themselves in conversation and further encourage person-centered care. The triangulation of data and the inclusion of both quantitative and qualitative data allows for extensive reports on the effectiveness of the training. It also allows us to report on facilitators and barriers for future interventions which seek to enhance the use of SDM in ACP conversations. One limitation is that enrollment in the training is voluntary: wards which are not motivated are not likely to participate. Since this is a single-blind trial (due to the control group receiving the training only at a later date) there should be no intentional bias on the part of the participants. Another limitation concerns the upper limit of 6 participants for each ward. Ideally, every staff member who engages in daily conversation with residents and their families should receive the training, thus truly achieving a ward-level approach to ACP. Since two wards receive training together, we set a maximum of 12 participants per group so as not to hinder interaction and assimilation. Participants are also stimulated to disseminate the training components and materials to other staff members and/or incorporate elements thereof into their own practice policy.

This study describes a hands-on, multi-level training approach to improve the level of SDM in ACP conversations. By providing tools to ward staff, engaging the management and informing residents and their families, ‘We DECide optimized’ aims to decrease evidence-based barriers and to provide all stakeholders incentives to engage in conversations about (care) preferences in an informative and participatory manner. The intervention also provides insight into policymakers and researchers concerning the long-term implementation barriers and facilitators for interventions which seek to enhance SDM.

## Additional files

Additional file 1: SACP: Dutch version of the questionnaire. (DOCX 32 kb)

Additional file 2: SACP: English version of the questionnaire. (DOCX 20 kb)

Additional file 3: Interview form: Dutch version of the interview form. (DOCX 12 kb)

Additional file 4: Interview form: English version of the interview form. (DOCX 14 kb)

Additional file 5: ACCENT: Dutch version of the questionnaire. (DOC 127 kb)

Additional file 6: ACCENT: English version of the questionnaire. (DOC 128 kb)

## Abbreviations

ACP: Advance Care Planning
SDM: Shared Decision Making
