# Supplementary material for: ‘We DECide optimized’ - training nursing home staff in shared decision-making skills for advance care planning conversations in dementia care: protocol of a pretest-posttest cluster randomized trial
Source: BMC Geriatr. 2019 Feb 4;19:33. doi: 10.1186/s12877-019-1044-z (PMC6360673; doi:10.1186/s12877-019-1044-z)
Supplement: Supplementary file 1 — SACP: Dutch version of the questionnaire. (DOCX 32 kb) [file 12877_2019_1044_MOESM1_ESM.docx]

***Vroegtijdige zorgplanning in het woonzorgcentrum: ondersteuning en opvolging (SACP)***

*Demografische gegevens*

Wat is uw naam?

Wat is uw geslacht?

Man

Vrouw

Wat is uw leeftijd?

Wat is uw hoogst behaalde diploma?

Secundair onderwijs

Hoger onderwijs niet-universitair

Hoger onderwijs universitair

Wat is de naam van het woonzorgcentrum waar u tewerkgesteld bent?

Wat is de naam van de afdeling waar u tewerkgesteld bent?

Wat is uw functie binnen dit woonzorgcentrum?

Zorgprofessional (zorgkundige, verpleegkundige, paramedicus,…)

Lid van het middenmanagement (hoofdverpleegkundige, referentiepersoon,…)

Lid van de raad van bestuur (directie, manager,…)

Voert u gesprekken over vroegtijdige zorgplanning met bewoners en/of naasten?

Ja

Nee

|  | u dit **belangrijk** vindt: | dit **gerealiseerd** is: |
| --- | --- | --- |
|  | 1= helemaal niet belangrijk  2= niet belangrijk  3= belangrijk en niet belangrijk  4= belangrijk  5= zeer belangrijk | 1= helemaal niet gerealiseerd  2= niet gerealiseerd  3= gerealiseerd en niet gerealiseerd  4= gerealiseerd  5= helemaal gerealiseerd |
| 1. De afdeling heeft een uitgewerkte visie op vroegtijdige zorgplanning. | **1 2 3 4 5** | **1 2 3 4 5** |
| 1. VZP is een vast punt op de agenda tijdens vergaderingen. | **1 2 3 4 5** | **1 2 3 4 5** |
| 1. De werking rond VZP wordt uitgedragen naar bewoners en naasten. | **1 2 3 4 5** | **1 2 3 4 5** |
| 1. Er wordt systematisch tijd vrij gemaakt om VZP te bespreken met bewoners en naasten. | **1 2 3 4 5** | **1 2 3 4 5** |
| 1. Er wordt geïnvesteerd in opleiding van het personeel om VZP te bespreken. | **1 2 3 4 5** | **1 2 3 4 5** |
| 1. Alle personeelsleden op de afdeling dragen bij tot VZP. | **1 2 3 4 5** | **1 2 3 4 5** |
| 1. Het management ondersteunt de werking rond VZP. | **1 2 3 4 5** | **1 2 3 4 5** |
| 1. Bewoners en naasten worden gestimuleerd om (levenseinde)zorg te bespreken. | **1 2 3 4 5** | **1 2 3 4 5** |

De volgende stellingen gaan over de opvolging en ondersteuning van vroegtijdige zorgplanning (VZP) op de afdeling.

Geef aan in welke mate:
