# Supplementary material for: ‘We DECide optimized’ - training nursing home staff in shared decision-making skills for advance care planning conversations in dementia care: protocol of a pretest-posttest cluster randomized trial
Source: BMC Geriatr. 2019 Feb 4;19:33. doi: 10.1186/s12877-019-1044-z (PMC6360673; doi:10.1186/s12877-019-1044-z)
Supplement: Supplementary file 2 — SACP: English version of the questionnaire. (DOCX 20 kb) [file 12877_2019_1044_MOESM2_ESM.docx]

***Advance care planning in residential care facilities: support and follow-up (SACP)***

*Demographic data*

What is your name?

What is your sex?

Male

Female

What is your age?

What is your highest level of education?

Secondary education

Higher education (not university)

Higher education (university)

What is the name of the residential care facility where you are employed?

What is the name of the department where you are employed?

What is your position at this residential care facility?

Care professional (care provider, nurse, paramedic, etc.)

Middle management professional (head nurse, reference person, etc.)

Member of the board of directors (executive, manager, etc.)

Do you participate in discussions about advance care planning with residents and/or the persons close to them?

Yes

No

|  | The extent to which you think this is **important**: | The extent to which this has been **achieved**: |
| --- | --- | --- |
|  | 1= not important at all  2= not important  3= neither important nor unimportant  4= important  5= very important | 1= not achieved at all  2= not achieved  3= neither achieved nor not achieved  4= achieved  5= fully achieved |
| 1. The department has developed a concrete vision with regard to advance care planning. | **1 2 3 4 5** | **1 2 3 4 5** |
| 1. ACP is a fixed item on the agenda during meetings. | **1 2 3 4 5** | **1 2 3 4 5** |
| 1. Operations with regard to ACP are communicated to residents and the persons close to them. | **1 2 3 4 5** | **1 2 3 4 5** |
| 1. Time is systematically made available to discuss ACP with residents and the persons close to them. | **1 2 3 4 5** | **1 2 3 4 5** |
| 1. We invest in training our staff to discuss ACP. | **1 2 3 4 5** | **1 2 3 4 5** |
| 1. All staff members at the department contribute to ACP. | **1 2 3 4 5** | **1 2 3 4 5** |
| 1. The management supports all operations related to ACP. | **1 2 3 4 5** | **1 2 3 4 5** |
| 1. Residents and the persons close to them are encouraged to discuss (end-of-life) care. | **1 2 3 4 5** | **1 2 3 4 5** |

The following statements concern follow-up and support with regard to advance care planning (ACP) at the department.

Please indicate the extent to which the following statements apply to you:
