# Supplementary material for: ‘We DECide optimized’ - training nursing home staff in shared decision-making skills for advance care planning conversations in dementia care: protocol of a pretest-posttest cluster randomized trial
Source: BMC Geriatr. 2019 Feb 4;19:33. doi: 10.1186/s12877-019-1044-z (PMC6360673; doi:10.1186/s12877-019-1044-z)
Supplement: Supplementary file 3 — Interview form: Dutch version of the interview form. (DOCX 12 kb) [file 12877_2019_1044_MOESM3_ESM.docx]

Telefonisch interview – 3 maanden follow up

1) Wat is de impact van de training na drie maanden?

2) Zijn er sinds de opleiding veranderingen gebeurd op het vlak van vroegtijdige zorgplanning? Zo ja, welke? Zijn er plannen om veranderingen rond VZP door te voeren in de komende periode?

3) Hoe werd het materiaal ingezet? Hoe werd het gebruik ervan ervaren door de deelnemers? En wat waren de reacties van de bewoners met dementia en hun familieleden?

4) Resterende werkpunten voor de afdeling?

5) Resterende werkpunten voor de deelnemers?

6) Toetsen naar:

- grootte van de groepen

- locatie

- tijdsduur

- ervaringen met de trainer

- aanwezigheid andere afdeling

- onderdelen training

7) Wat neemt u vooral mee uit de training?

8) Hoe zou de training nog verbeterd kunnen worden?

9) Heeft de training op enige manier een negatieve impact gehad op de afdeling?

10) Heeft u nog vragen?

Telefonisch interview – 9 maanden follow up

1) Wat is de impact van de training na 9 maanden?

2) Zijn er sinds de opleiding veranderingen gebeurd op het vlak van vroegtijdige zorgplanning? Zo ja, welke? Zijn er plannen om veranderingen rond VZP door te voeren in de komende periode?

3) Hoe werd het materiaal verder ingezet? Hoe werd het gebruik ervan ervaren door de deelnemers? En wat waren de reacties van de bewoners met dementia en hun familieleden?

4) Hoe werden de werkpunten die besproken werden tijdens het vorige telefonisch overleg in de voorbije maanden aangepakt?

5) Resterende werkpunten voor de afdeling?

6) Resterende werkpunten voor de deelnemers?

7) Welke inzichten neemt u mee uit de training na deze 9 maanden?

8) Heeft u nog vragen?
