# Supplementary material for: ‘We DECide optimized’ - training nursing home staff in shared decision-making skills for advance care planning conversations in dementia care: protocol of a pretest-posttest cluster randomized trial
Source: BMC Geriatr. 2019 Feb 4;19:33. doi: 10.1186/s12877-019-1044-z (PMC6360673; doi:10.1186/s12877-019-1044-z)
Supplement: Supplementary file 4 — Interview form: English version of the interview form. (DOCX 14 kb) [file 12877_2019_1044_MOESM4_ESM.docx]

Interview questions: 3 months follow-up

1) What is the impact of the training after 3 months?

2) Have any changes occurred in the advance care planning policy since the training? If so, which? If not, are there any plans to update it?

3) Has the material been put to use? What were the experiences of professionals in using it? And how did the residents with dementia and their relatives react to it?

4) What are the remaining points for improvement at the ward level?

5) What are the remaining points for improvement at the individual level?

6) What are your thoughts on:

- The size of the training group

- The location

- The duration of the training

- The trainer

- The training components

- The presence of a different nursing home ward

7) What were your take-home messages after attending both workshops?

8) How could the training be improved?

9) Did the training have a negative impact in any way?

10) Do you have any further questions?

Interview questions: 9 months follow-up

1) What is the impact of the training after 9 months?

2) Have any changes occurred in the advance care planning policy? If so, which? If not, are there any plans to update it?

3) Has the material been put to further use? What were the experiences of professionals in using it? And how did the residents with dementia and their relatives react to it?

4) How were the points for improvement, which were discussed during the 3 months follow-up, addressed during the past 6 months?

5) What are the remaining points for improvement at the ward level?

6) What are the remaining points for improvement at the individual level?

7) Looking back on the training after 9 months, what will you remember the most?

8) Do you have any further questions?
