# Supplementary material for: ‘We DECide optimized’ - training nursing home staff in shared decision-making skills for advance care planning conversations in dementia care: protocol of a pretest-posttest cluster randomized trial
Source: BMC Geriatr. 2019 Feb 4;19:33. doi: 10.1186/s12877-019-1044-z (PMC6360673; doi:10.1186/s12877-019-1044-z)
Supplement: Supplementary file 5 — ACCENT: Dutch version of the questionnaire. (DOC 127 kb) [file 12877_2019_1044_MOESM5_ESM.doc]

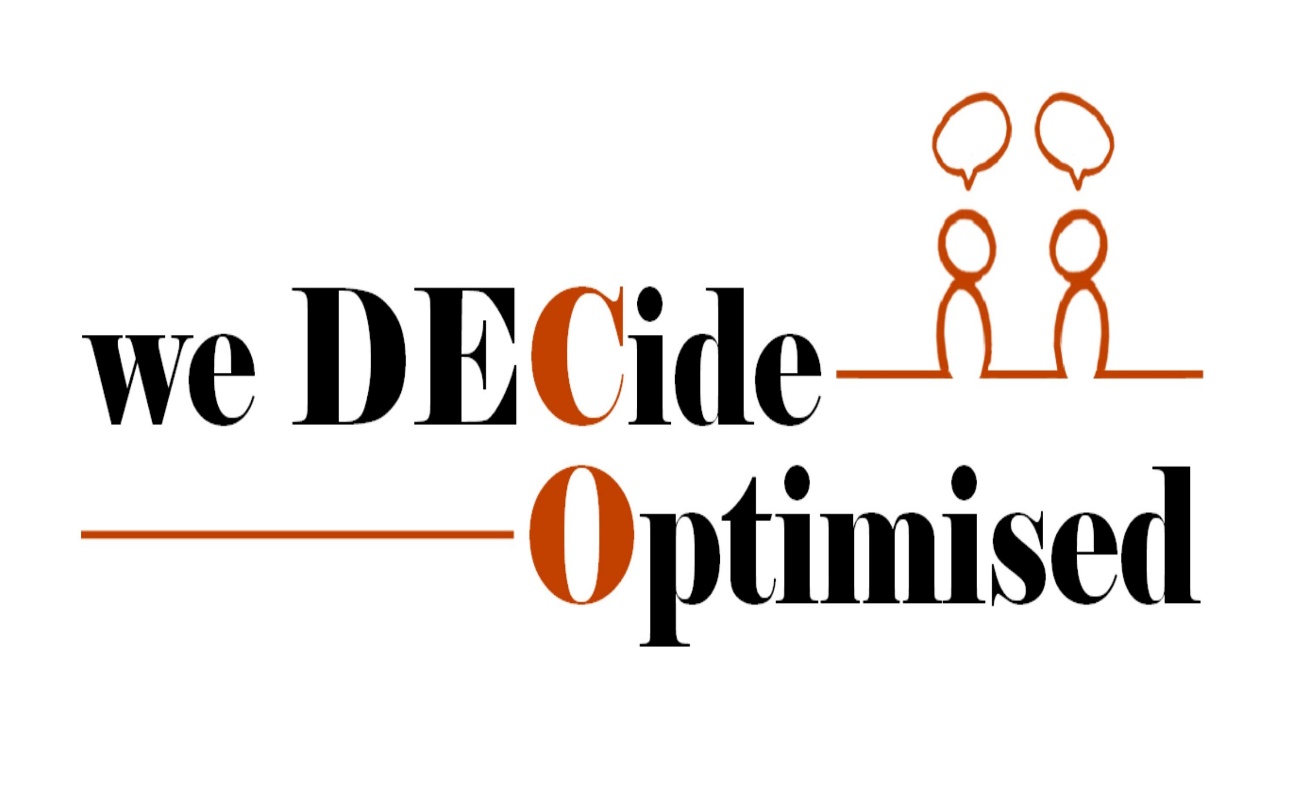


**ACCENT**

Naam woonzorgcentrum en afdeling

De volgende uitspraken hebben betrekking op uw beoordeling van de teamvergadering tussen zorgverleners over vroegtijdige zorgplanning bij personen met dementie in de woonzorgcentra.

Kruis aan in hoeverre elke uitspraak van toepassing is.

**1. Het perspectief van de bewoner en/of naasten staat centraal.**

| Helemaal niet  van toepassing | Niet  van toepassing | Tussenin | Wel  van toepassing | Helemaal  van toepassing |
| --- | --- | --- | --- | --- |
| □ | □ | □ | □ | □ |
|  |  |  |  |  |

**2. In het team worden de voor- en nadelen van één of meerdere opties gewikt en gewogen.**

| Helemaal niet  van toepassing | Niet  van toepassing | Tussenin | Wel  van toepassing | Helemaal  van toepassing |
| --- | --- | --- | --- | --- |
| □ | □ | □ | □ | □ |

**3. Een gespreksleider bewaakt dat verschillende meningen tot hun recht komen.**

| Helemaal niet  van toepassing | Niet  van toepassing | Tussenin | Wel  van toepassing | Helemaal  van toepassing |
| --- | --- | --- | --- | --- |
| □ | □ | □ | □ | □ |

**4. Aanwezigen nemen gelijkwaardig deel aan het overleg.**

| Helemaal niet  van toepassing | Niet  van toepassing | Tussenin | Wel  van toepassing | Helemaal  van toepassing |
| --- | --- | --- | --- | --- |
| □ | □ | □ | □ | □ |

**5. Het team formuleert concrete aandachtspunten voor de besluitvorming met bewoner en/of naasten.**

| Helemaal niet  van toepassing | Niet  van toepassing | Tussenin | Wel  van toepassing | Helemaal  van toepassing |
| --- | --- | --- | --- | --- |
| □ | □ | □ | □ | □ |

Opmerkingen:
