# Supplementary material for: ‘We DECide optimized’ - training nursing home staff in shared decision-making skills for advance care planning conversations in dementia care: protocol of a pretest-posttest cluster randomized trial
Source: BMC Geriatr. 2019 Feb 4;19:33. doi: 10.1186/s12877-019-1044-z (PMC6360673; doi:10.1186/s12877-019-1044-z)
Supplement: Supplementary file 6 — ACCENT: English version of the questionnaire. (DOC 128 kb) [file 12877_2019_1044_MOESM6_ESM.doc]

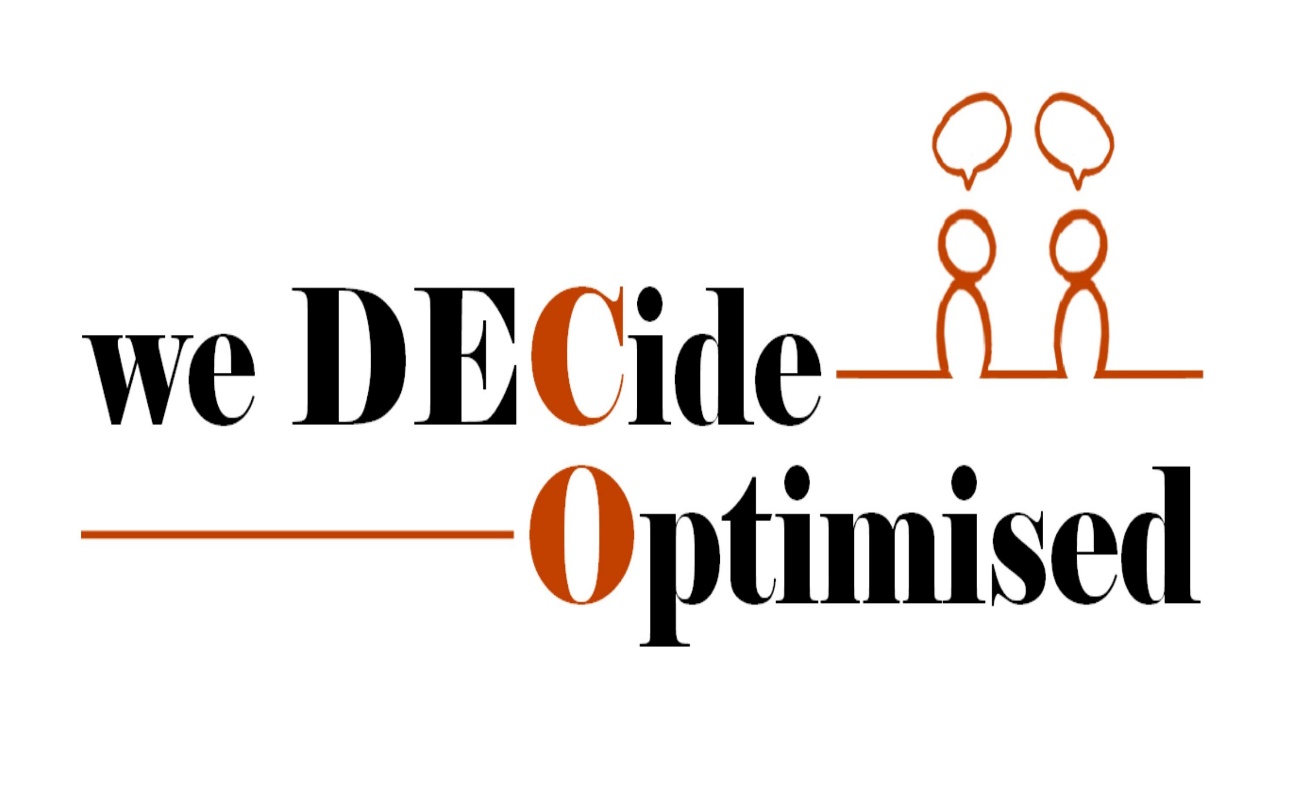


**ACCENT**

Name and department of residential care facility

The following statements are related to your assessment of the team meetings with care providers about advance care planning for inhabitants of residential care facilities who suffer from dementia.

Indicate the extent to which each statement is applicable to your situation.

**1. Priority is given to the point of view of the resident and/or the persons close to him/her.**

| Not at all  applicable | Not applicable | Partly applicable | Applicable | Fully  applicable |
| --- | --- | --- | --- | --- |
| □ | □ | □ | □ | □ |
|  |  |  |  |  |

**2. The pros and cons of one or several options are carefully taken into consideration by the team.**

| Not at all  applicable | Not applicable | Partly applicable | Applicable | Fully  applicable |
| --- | --- | --- | --- | --- |
| □ | □ | □ | □ | □ |

**3. A discussion leader monitors the discussions to ensure that all the opinions given are given equal attention.**

| Not at all  applicable | Not applicable | Partly applicable | Applicable | Fully  applicable |
| --- | --- | --- | --- | --- |
| □ | □ | □ | □ | □ |

**4. Everyone present participates in the meeting on equal terms.**

| Not at all  applicable | Not applicable | Partly applicable | Applicable | Fully  applicable |
| --- | --- | --- | --- | --- |
| □ | □ | □ | □ | □ |

**5. The team formulates concrete points for attention with regard to decisions with the residents and/or the persons close to them.**

| Not at all  applicable | Not applicable | Partly applicable | Applicable | Fully  applicable |
| --- | --- | --- | --- | --- |
| □ | □ | □ | □ | □ |

Remarks:
